# The Role of Ayurveda in Chronic Disease Management: A Review of Clinical Evidence

**DOI:** 10.7759/cureus.105199

**Published:** 2026-03-13

**Authors:** Avinash D Karambhe, Sonam V Surkar, Amruta M Deokar, Ganesh Hedaoo, Omprakash Ramkrushna Gulhane, Sharayu S Dingalwar

**Affiliations:** 1 Department of Kaumarbhritya (Balrog), Bhausaheb Mulak Ayurved College, Nagpur, IND; 2 Department of Kaumarbhritya (Balrog), Maharashtra University of Health Sciences, Nashik, IND; 3 Department of Kayachikitsa, SKS Ayurvedic Medical College and Hospital, Mathura, IND; 4 Department of Kaumarbhritya, Mandsaur Institute of Ayurved Education and Research, Mandsaur University, Mandsaur, IND; 5 Department of Sharir Kriya, Maharashtra University of Health Sciences, Nashik, IND; 6 Department of Swasthavritta, Bhausaheb Mulak Ayurved Mahavidyalaya, Nagpur, IND; 7 Department of Swasthavritta and Yoga, Bhausaheb Mulak Ayurved Mahavidyalaya, Nagpur, IND; 8 Department of Swasthavritta and Yoga, Maharashtra University of Health Sciences, Nashik, IND

**Keywords:** ayurveda, chronic disease management, clinical evidence, integrative medicine, non-communicable diseases

## Abstract

Chronic non-communicable diseases account for the majority of global mortality and contribute substantially to long-term disability and healthcare expenditure worldwide, necessitating sustained, multidimensional management strategies beyond symptomatic control. Ayurveda, a traditional system of medicine based on holistic and individualized care, has gained increasing attention as a complementary approach for chronic disease management. This review synthesizes clinical evidence published between 2015 and 2025 to evaluate the role of Ayurvedic interventions across major chronic disease categories, including metabolic, cardiovascular, musculoskeletal, respiratory, gastrointestinal, and neurological disorders. A comprehensive literature search of major electronic databases identified randomized controlled trials, controlled clinical studies, and observational research assessing therapeutic outcomes, safety, and methodological quality. The reviewed studies demonstrate consistent trends toward improvement in symptom severity, functional outcomes, biochemical and inflammatory markers, and patient-reported quality of life, particularly when Ayurvedic therapies are used alongside conventional medical care. Interventions based on Ayurvedic principles, such as metabolic regulation, lifestyle modification, Panchakarma (bio-purification procedures), and Rasayana (rejuvenative therapy), appear to exert anti-inflammatory, antioxidant, immunomodulatory, and adaptogenic effects relevant to chronic disease pathophysiology. The evidence base is constrained by methodological heterogeneity, small sample sizes, variable intervention protocols, and limited long-term safety data. While current findings support the integrative potential of Ayurveda in chronic disease management, further rigorously designed, large-scale clinical trials with standardized methodologies are required to strengthen evidence and support its rational incorporation into contemporary healthcare systems.

## Introduction and background

Chronic diseases represent a significant and increasing global health burden and contribute substantially to morbidity, mortality, and healthcare spending worldwide, causing at least 43 million deaths in 2021, equivalent to approximately 75% of all non-pandemic-related deaths globally, with 73% of these deaths occurring in low- and middle-income countries according to the World Health Organization in 2025 [1]. These non-communicable diseases are characterized by long-term progression, multifactorial pathophysiology, and the requirement for sustained management strategies [2]. Their rising prevalence is closely associated with demographic transitions, urbanization, sedentary behavior, dietary shifts, and increasing life expectancy [3]. Consequently, health systems globally face the dual challenge of limiting disease progression while preserving long-term functional capacity and quality of life [4].

Modern biomedical management of chronic illnesses has resulted in major advances in diagnosis, pharmacotherapy, and supportive care [5]. Long-term treatment, however, is frequently associated with adverse drug reactions, polypharmacy, declining adherence, and escalating costs, with global health expenditure reaching approximately US$ 8.3 trillion in 2019, representing about 10% of global gross domestic product, as reported by the World Health Organization [6]. In many chronic conditions, therapeutic strategies primarily target symptom suppression or biochemical endpoints, often with limited emphasis on individualized regulation of systemic homeostasis or long-term disease modification [7]. Moreover, even under optimal conventional care, a substantial proportion of patients continue to experience residual symptoms and impaired well-being; for example, approximately 30% of patients with major depressive disorder in the STAR*D trial failed to achieve remission despite sequential evidence-based treatments [8]. These limitations have contributed to increasing interest in complementary and integrative approaches that incorporate individualized care, lifestyle modification, psychosocial determinants, and patient-reported outcomes alongside conventional biomedical targets [9].

**Figure 1:**
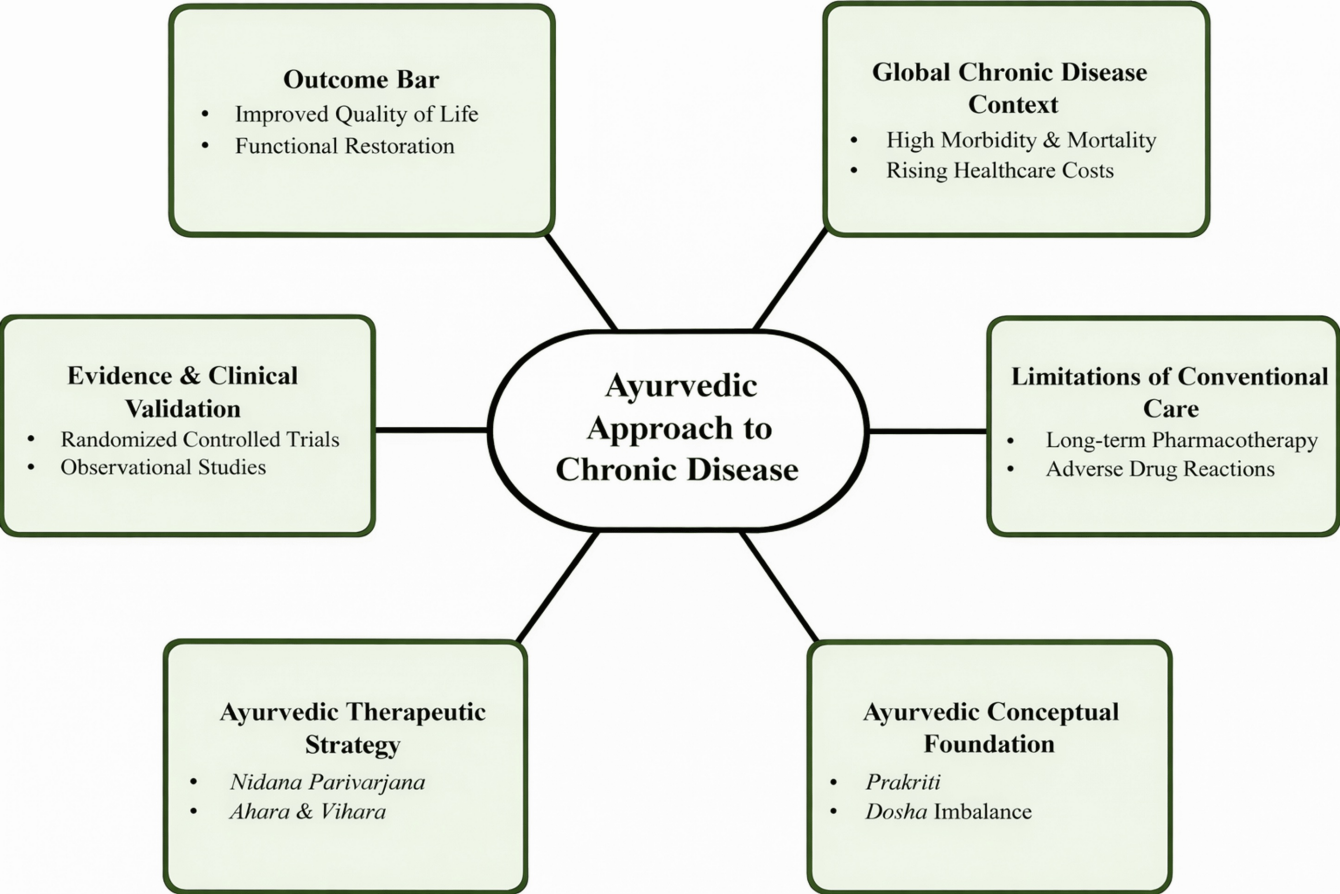
Conceptual framework of the Ayurvedic approach to chronic disease management This figure shows the relationship between the global chronic disease burden, limitations of conventional care, Ayurvedic conceptual foundations, therapeutic strategies, and supporting clinical evidence, highlighting how these components collectively contribute to functional restoration and improved quality of life. This image was created using Microsoft PowerPoint (Microsoft Corp., Redmond, WA, USA).

Ayurveda is a traditional medical system with a history spanning several millennia and presents a structured framework for understanding chronic disease through systemic regulation rather than isolated pathology [10]. Health is conceptualized as a dynamic equilibrium among physiological, psychological, and environmental domains [11]. Core principles include *Prakriti* (individual constitutional profile), *Dosha* (functional regulatory systems), *Agni* (metabolic and transformative processes), and *Ama* (pathological metabolic by-products arising from impaired processing) [12]. *Prakriti* reflects an individual’s inherent constitutional makeup determined by genetic, metabolic, and physiological traits, while the three *doshas *(*Vata*, *Pitta*, and *Kapha*) represent functional regulatory patterns governing movement, transformation, and structural stability within the body. From a biomedical interpretative perspective, *Doshas* may be viewed as functional regulatory networks governing neuroendocrine, metabolic, and inflammatory processes, while *Agni* reflects metabolic efficiency and *Ama* corresponds conceptually to dysregulated metabolic or inflammatory intermediates contributing to tissue dysfunction. Chronic diseases (*Chirakari Vyadhi*) are described as conditions arising from sustained physiological imbalance driven by improper diet, lifestyle, psychosocial stress, and progressive metabolic disturbance [13]. This conceptualization parallels contemporary models of chronic disease as dynamic, multifactorial processes involving systemic dysregulation rather than isolated pathological events [14]. As illustrated in Figure 1, Ayurveda proposes an integrative framework for long-term chronic disease management.

The Ayurvedic approach to chronic disease integrates preventive, therapeutic, and rehabilitative strategies [15]. Management protocols emphasize *Nidana Parivarjana* (avoidance of etiological factors), personalized dietary and lifestyle modification (*Ahara* and *Vihara*), herbal and herbo-mineral pharmacological interventions, bio-purificatory procedures (*Panchakarma*), and rejuvenative therapies (*Rasayana*) [16]. Equal emphasis is placed on mental health and psychosomatic balance, recognizing bidirectional interactions between chronic physical illness and psychological well-being [17]. Such multidimensional strategies are particularly relevant in chronic disorders requiring long-term modulation of disease activity and functional optimization [18].

Over recent decades, increasing efforts have been made to evaluate Ayurvedic interventions using modern clinical research methodologies [19]. Randomized controlled trials, controlled clinical studies, observational investigations, and integrative care models have examined the role of Ayurveda in type 2 diabetes mellitus, hypertension, osteoarthritis, rheumatoid arthritis, bronchial asthma, gastrointestinal disorders, and neuropsychiatric conditions [20]. Reported outcomes include improvements in symptom scores, functional indices, and selected biochemical and inflammatory markers [21]. For example, controlled clinical studies in type 2 diabetes have reported reductions in fasting blood glucose and glycated hemoglobin following standardized Ayurvedic interventions, while trials in osteoarthritis have demonstrated improvements in pain and functional assessment scales compared with baseline or control groups. Additionally, experimental and clinical investigations suggest anti-inflammatory, antioxidant, immunomodulatory, and adaptogenic effects that are mechanistically relevant to chronic disease pathophysiology [22].

Despite this expanding literature, the clinical evidence base remains heterogeneous in study design, methodological rigor, sample size, intervention standardization, outcome measures, and duration of follow-up. Inconsistent reporting of adverse events and variability in internal validity limit the strength of generalized conclusions. Moreover, existing publications are frequently disease-specific or formulation-specific, making it difficult to derive an integrated perspective across chronic disease domains. A structured and critical synthesis of available clinical evidence across major chronic disease categories is therefore necessary to clarify therapeutic scope, identify methodological strengths and weaknesses, and delineate areas requiring more rigorous investigation.

This narrative review provides a comprehensive and critical synthesis of human clinical studies evaluating Ayurvedic interventions across metabolic, cardiovascular, musculoskeletal, respiratory, gastrointestinal, and neuropsychiatric chronic diseases, with emphasis on therapeutic outcomes, safety considerations, and methodological quality.

Objectives of the review

The present review examines and synthesizes human clinical studies evaluating Ayurvedic interventions in the management of chronic diseases to assess therapeutic outcomes, safety profiles, and methodological rigor across diverse disease categories. Specifically, the review aims to (1) summarize disease-specific clinical outcomes reported in controlled and observational studies, (2) critically appraise study design quality and sources of bias, (3) evaluate safety reporting and tolerability data, and (4) identify research gaps and priorities for future large-scale, methodologically robust investigations. By integrating evidence across multiple domains, this review seeks to support informed clinical interpretation and guide rational integration of Ayurveda within contemporary chronic disease management frameworks.

Methodology

Search Strategy

A comprehensive literature search was conducted to identify human clinical studies evaluating Ayurvedic interventions in the management of chronic diseases. The search covered publications from January 2015 to December 2025 and was performed using major electronic databases, including PubMed, Scopus, Web of Science, Google Scholar, the AYUSH Research Portal, and the Cochrane Library. Relevant keywords and Medical Subject Headings (MeSH) were used individually and in combination, including “Ayurveda”, “Ayurvedic medicine”, “chronic disease”, “non-communicable diseases”, “clinical trial”, “randomized controlled trial”, and “integrative medicine”, and disease-specific terms such as diabetes, hypertension, arthritis, asthma, gastrointestinal disorders, and neurodegenerative diseases. Boolean operators (AND/OR) were applied to construct search strings (e.g., Ayurveda AND chronic disease AND clinical trial), and database-specific adaptations were implemented where appropriate. An example of the search strategy used in PubMed was (“Ayurveda” OR “Ayurvedic medicine”) AND (“chronic disease” OR “non-communicable diseases”) AND (“clinical trial” OR “randomized controlled trial”). Similar search strings were adapted for other databases with adjustments to database-specific indexing terms where necessary to improve search reproducibility. Reference lists of relevant publications were also manually screened to identify additional eligible studies.

This review was conducted as a narrative synthesis. The search strategy was intentionally broad to capture diverse clinical evidence rather than to perform quantitative meta-analysis. Although the review did not follow PRISMA guidelines for systematic reviews, the study identification, screening, and eligibility assessment steps were documented to improve methodological transparency and reproducibility. The review did not employ formal evidence grading systems or pooled statistical analysis.

Eligibility Criteria

Studies were considered eligible if they involved human participants, were published in English, evaluated Ayurvedic interventions for chronic disease conditions, and reported measurable clinical outcomes, biochemical parameters, functional indices, or quality-of-life measures. Randomized controlled trials, controlled clinical trials, observational studies, and pilot clinical studies were included. Studies were excluded if they were conducted in animals or in vitro models, were conference abstracts without full-text availability, were published in languages other than English, or did not clearly specify measurable clinical outcomes.

Given the narrative nature of the review, study selection prioritized clinical relevance, methodological clarity, and contribution to understanding therapeutic effectiveness rather than strict quantitative comparability.

Study Selection and Data Extraction

Titles and abstracts were screened for relevance, followed by full-text evaluation of potentially eligible studies. Duplicate records were removed prior to screening. The database search yielded multiple records across the selected databases, and after removal of duplicates and screening of titles and abstracts, full-text articles were assessed for eligibility. Studies were included only if they met all predefined eligibility criteria after full-text evaluation. A total of 47 studies met the inclusion criteria and were included in the final narrative synthesis. Data extraction focused on study design, sample characteristics and disease stage where reported, type of Ayurvedic intervention (including herbal formulations, herbo-mineral preparations, Panchakarma procedures, lifestyle-based interventions, and integrative care models), treatment duration, comparator type (placebo, standard therapy, or adjunctive use), outcome measures, magnitude of reported clinical effects, and documented adverse events.

Appraisal and Synthesis

Methodological quality and potential sources of bias were appraised qualitatively by examining the adequacy of randomization procedures, presence and appropriateness of control groups, blinding where applicable, completeness of outcome reporting, and transparency in adverse event documentation. This qualitative appraisal was intended to identify potential sources of bias and methodological limitations within individual studies rather than to assign quantitative risk-of-bias scores. No formal risk-of-bias scoring tool or quantitative grading framework was applied. The synthesis was descriptive and thematic, aiming to identify patterns of clinical effectiveness, comparator context, safety trends, and methodological limitations across metabolic, cardiovascular, musculoskeletal, respiratory, gastrointestinal, and neuropsychiatric chronic diseases rather than to generate pooled effect estimates.

## Review

Conceptual framework of chronic disease in Ayurveda

Ayurveda presents a systemic conceptual framework for understanding chronic disease that emphasizes long-term physiological imbalance rather than isolated pathology [23]. Classical texts describe chronic diseases as *Chirakari Vyadhi*, conditions that develop gradually due to sustained exposure to etiological factors and persistent internal dysregulation [4]. Rather than focusing on localized organ pathology, Ayurveda conceptualizes chronic illness as the cumulative consequence of impaired digestion, metabolism, tissue nourishment, and elimination processes [24]. This systems-oriented perspective aligns with contemporary biomedical models that recognize chronic diseases as multifactorial conditions influenced by metabolic dysfunction, lifestyle factors, environmental exposures, and psychosocial stressors [13].

A central component of Ayurvedic pathogenesis is *Dosha Dushti*, denoting functional imbalance among *Vata*, *Pitta*, and *Kapha*, which regulate physiological processes. *Vata* governs movement and neural activity, including circulation, respiration, and nerve signaling; *Pitta* regulates metabolism and biochemical transformation, including digestion and thermoregulation; and *Kapha* maintains structural stability, lubrication, and aspects of immune resilience. Disturbance of these regulatory principles is understood to disrupt systemic homeostasis and initiate progressive disease processes [25]. Sustained *Dosha* imbalance may lead to *Dhatu Kshaya*, referring to progressive tissue degeneration and functional decline [7], a phenomenon observed in chronic disorders characterized by weakness, structural deterioration, or persistent dysfunction [26].

Another key pathological construct is *Ama*, described as incompletely metabolized or improperly processed biological by-products arising from impaired digestive and metabolic efficiency [12]. *Ama* is considered to obstruct physiological channels (*Srotas*), promote inflammatory processes, and impair immune regulation, thereby contributing to chronicity [27]. Impaired *Agni*, representing deficient metabolic transformation, is regarded as a central driver of both *Ama* accumulation and defective tissue nourishment, forming a core element in chronic disease development [6].

**Figure 2:**
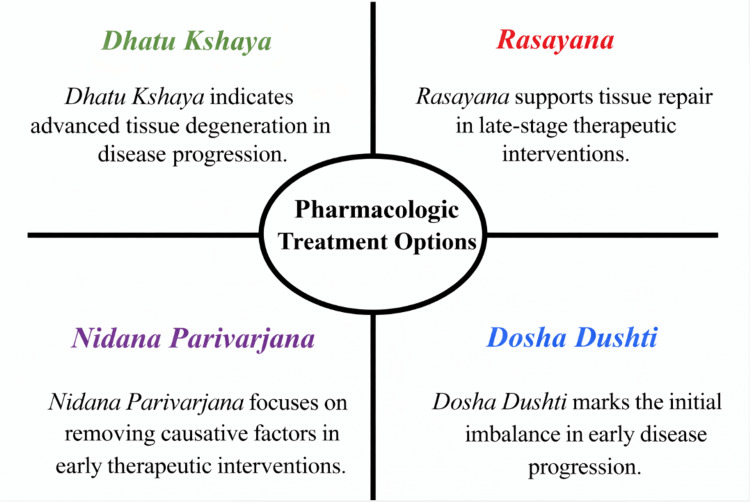
Staged Ayurvedic pathogenesis of chronic disease and targeted therapeutic interventions This figure illustrates the transition from early *dosha* imbalance (*dosha dushti*) to advanced tissue degeneration (*dhatu kshaya*), highlighting how interventions, such as *nidana parivarjana* and *rasayana*, are applied at different stages to prevent progression and support tissue restoration. This image was created using Microsoft PowerPoint (Microsoft Corp., Redmond, WA, USA).

Therapeutic strategies in Ayurveda are derived directly from this etiopathogenic framework and are implemented through a staged, individualized approach [28]. *Nidana Parivarjana*, or the removal of causative factors, forms the foundation of management and involves modifying diet, lifestyle, and behavioral contributors to disease progression [15]. *Shodhana* therapies, including *Panchakarma* procedures, are employed to eliminate accumulated *Doshas* and *Ama* in selected patients based on disease stage and physiological status, whereas *Shamana* therapies aim to restore balance through herbal formulations, dietary regulation, and lifestyle adjustments [16]. *Rasayana* interventions are incorporated to support tissue repair, immune competence, and functional recovery [18]. This integrated model emphasizes long-term systemic regulation rather than short-term symptomatic control [20]. The staged relationship between pathogenesis and therapeutic intervention is illustrated in Figure 2.

Ayurvedic interventions in metabolic disorders (diabetes and obesity)

Metabolic disorders, particularly diabetes mellitus and obesity, represent a major component of the global chronic disease burden, with 537 million adults living with diabetes in 2021 and 890 million adults living with obesity in 2022; additionally, 43% of adults worldwide are overweight and 16% are living with obesity [29]. Within Ayurveda, diabetes (*Madhumeha*) is classified under *Prameha*, a group of metabolic disorders associated with *Kapha* predominance, impaired *Agni* (metabolic regulation), and progressive disturbance of tissue metabolism [8]. Obesity (*Sthaulya*) is described as pathological accumulation and dysfunction of *Meda Dhatu* (adipose tissue), often linked to reduced metabolic efficiency and inflammatory tendencies [30]. These constructs demonstrate conceptual convergence with biomedical mechanisms such as insulin resistance, dyslipidemia, adipose tissue dysfunction, and chronic low-grade inflammation [14].

Recent clinical investigations have increasingly evaluated Ayurvedic interventions using standardized biomedical outcome measures, including fasting blood glucose, glycated hemoglobin (HbA1c), lipid profiles, inflammatory markers, and anthropometric indices [31]. Controlled trials of *Nisha Amalaki*, a formulation combining *Curcuma longa *(turmeric) and *Emblica officinalis* (amla), have reported reductions in fasting and postprandial blood glucose, with short-term decreases in fasting glucose, ranging approximately between 10% and 25% in selected study populations [32]. These effects are generally modest compared with standard oral hypoglycemic agents, such as metformin, but may be clinically relevant when used as adjunctive therapy. Proposed mechanisms include antioxidant and anti-inflammatory activities, modulation of inflammatory cytokines, improvement in insulin sensitivity, and inhibition of carbohydrate-digesting enzymes, such as α-amylase and α-glucosidase.

*Gymnema sylvestre* (*Gudmar*) has demonstrated potential glycemic improvements and enhancement of insulin responsiveness in small clinical studies, while *Triphala* has been associated with favorable changes in lipid parameters, body weight indices, and oxidative stress markers [17,33]. Evidence for Ayush-64 in metabolic contexts remains exploratory, with small-scale trials suggesting possible immunomodulatory and metabolic effects; however, heterogeneity in study design, limited follow-up duration, and modest sample sizes constrain definitive interpretation [19].

Across metabolic interventions, the comparator context varies substantially, with studies evaluating monotherapy, adjunctive use alongside conventional pharmacotherapy, or lifestyle-integrated regimens. Although several trials report statistically significant improvements in glycemic and lipid parameters, reproducibility and long-term outcome data remain limited. Greater standardization of formulations, adequately powered multicentric randomized trials, and long-term safety monitoring are required to determine comparative effectiveness and clinical positioning within integrative metabolic care frameworks.

Beyond pharmacological interventions, Ayurveda emphasizes structured lifestyle and dietary modification as foundational components of metabolic management [21]. Personalized dietary regulation, meal timing, structured physical activity, and stress modulation constitute *Ahara*-*Vihara* strategies integrated into therapeutic plans [22]. Available clinical evidence suggests that combined lifestyle and herbal approaches may enhance adherence and contribute to metabolic risk reduction, though independent effect attribution remains methodologically challenging [25]. This integrated behavioral-metabolic orientation aligns with contemporary preventive models but requires more rigorous longitudinal evaluation.

Role of Ayurveda in cardiovascular diseases

Cardiovascular diseases, including hypertension, ischemic heart disease, and dyslipidemia, have increasingly been examined within Ayurvedic clinical research [34]. Ayurvedic pathophysiology attributes these disorders to *Vata* imbalance, obstruction of physiological channels, impaired circulation, and compromised tissue nourishment, often influenced by metabolic disturbance and psychological stress [11]. These concepts broadly parallel biomedical mechanisms involving endothelial dysfunction, autonomic imbalance, oxidative stress, and systemic inflammation [27].

Clinical investigations have evaluated several individual herbs and classical formulations for cardiometabolic modulation. *Terminalia arjuna* (Arjuna) is among the most studied agents, with trials reporting reductions in systolic and diastolic blood pressure, improvements in lipid parameters, and changes in selected measures of cardiac function, including left ventricular performance indices in specific patient populations [35]. *Withania somnifera* (Ashwagandha) has been examined for stress-related cardiovascular modulation, while *Allium sativum *(garlic) and *Commiphora mukul* (Guggulu) preparations have demonstrated lipid-lowering and anti-inflammatory effects in controlled settings [36]. Some studies report improvements in exercise tolerance and symptom burden in chronic stable angina when Ayurvedic interventions are used adjunctively.

However, most cardiovascular studies involve relatively small cohorts, short intervention durations, and variable comparator frameworks. Many trials assess Ayurvedic therapies as adjuncts to conventional treatment rather than as standalone alternatives [37]. Reported benefits therefore frequently reflect additive or supportive effects rather than replacement of standard pharmacotherapy. Variability in formulation standardization and outcome measures limits direct cross-study comparison.

Taken collectively, available evidence suggests that selected Ayurvedic interventions may influence lipid regulation, blood pressure control, oxidative stress, and inflammatory pathways relevant to cardiovascular disease. Nonetheless, methodological heterogeneity and limited large-scale randomized data necessitate cautious interpretation. Robust, long-term, multicentric trials with standardized preparations and clearly defined cardiovascular endpoints are required to determine the extent to which these interventions contribute to clinically meaningful risk reduction within integrative cardiovascular care models [38]. The principal Ayurvedic concepts, interventions, and reported clinical outcomes in cardiovascular disease are summarized in Table 1.

**Table 1:** Ayurvedic interventions and clinical evidence in cardiovascular diseases

Aspect	Ayurvedic perspective	Key clinical evidence	Reference
Disease focus	Hypertension, ischemic heart disease, dyslipidemia	Increasing clinical interest in Ayurvedic management of cardiovascular disorders	[34]
Etiopathogenesis	*Vata* imbalance, *Srotas* obstruction, impaired circulation, tissue nourishment defects	Conceptual alignment with endothelial dysfunction, inflammation, and oxidative stress	[27]
Core cardioprotective herb	*Terminalia arjuna* (Arjuna)	Reductions in systolic and diastolic blood pressure; decreased total cholesterol, LDL, and triglycerides; improved left ventricular ejection fraction (LVEF); enhanced exercise tolerance; reduced frequency of anginal episodes in chronic stable angina and mild-to-moderate heart failure	[36]
Anti-ischemic intervention	*Inula racemosa *(*Pushkarmool*)	Traditional and clinical use in ischemic symptoms and chest pain	[19]
Lipid-modulating therapy	Guggulu-based formulations	Lipid-lowering, anti-inflammatory, and antioxidant effects	[21]
Therapeutic positioning	Adjunctive use with conventional therapy	Additive benefits in symptom control and risk factor modulation	[7]
Evidence limitations	Methodological heterogeneity	Variability in study design, sample size, and rigor	[13]
Clinical relevance	Integrative care frameworks	Supports a complementary role in long-term cardiovascular disease management	[38]

Ayurvedic management of musculoskeletal and rheumatic disorders

Musculoskeletal and rheumatic disorders are major contributors to chronic pain, disability, and functional impairment worldwide [14]. Conditions such as osteoarthritis, rheumatoid arthritis, and chronic low back pain are characterized by persistent inflammation, progressive structural degeneration, joint stiffness, and reduced mobility [21]. Conventional management typically involves analgesics, non-steroidal anti-inflammatory drugs, corticosteroids, and disease-modifying agents; however, long-term use is frequently associated with adverse effects, incomplete disease modification, and variable functional outcomes [6]. These limitations have prompted interest in adjunctive strategies that prioritize sustained symptom control, preservation of joint function, and long-term tolerability [9].

Within Ayurvedic pathophysiology, musculoskeletal disorders are primarily attributed to an imbalance of *Vata*, the regulatory principle governing movement, neuromuscular coordination, and joint dynamics, often accompanied by accumulation of *Ama*, described as incompletely processed metabolic by-products that contribute to inflammatory obstruction and tissue dysfunction [12]. Classical entities such as *Sandhivata *(osteoarthritis) and *Amavata* (rheumatoid arthritis) are conceptualized as chronic conditions involving degeneration, inflammation, and obstruction of physiological channels (*Srotas*) [18]. This framework demonstrates conceptual convergence with biomedical models of cartilage degradation, synovial inflammation, immune dysregulation, and cytokine-mediated joint damage [27]. Therapeutic goals accordingly include reduction of *Ama*, pacification of *Vata*, attenuation of inflammation, and restoration of joint mobility through internal and external interventions [16].

Clinical investigations of *Panchakarma*-based therapies in rheumatic conditions have identified *Basti* as a frequently studied intervention for *Vata*-dominant presentations [23]. *Basti* involves rectal administration of medicated herbal decoctions or lipid-based preparations intended to exert systemic effects beyond bowel evacuation, differing from conventional medical enemas that primarily serve mechanical cleansing purposes. Controlled clinical studies report reductions in pain severity, joint stiffness, and functional disability scores in osteoarthritis and chronic low back pain populations following *Basti*-based regimens [25]. However, most trials involve modest sample sizes and short follow-up durations, and comparator frameworks vary between standard care, placebo, or pre-post single-arm designs.

Adjunctive external therapies, including *Abhyanga* (therapeutic oil massage) and *Swedana* (induced sudation), have been associated with improvements in joint mobility, muscle relaxation, and functional performance measures in selected studies [19]. Proposed mechanisms include enhanced local circulation, neuromuscular modulation, and reduction of inflammatory mediators, though mechanistic validation remains limited [30].

Integrative Ayurvedic treatment protocols combining internal herbal formulations with detoxification procedures in rheumatoid arthritis have demonstrated improvements in pain scores, morning stiffness duration, and inflammatory markers such as erythrocyte sedimentation rate and C-reactive protein in controlled clinical contexts [17]. These findings suggest potential adjunctive benefit; however, heterogeneity in study design, sample size, blinding procedures, and outcome standardization constrains definitive interpretation [26]. Current evidence does not support substitution of conventional disease-modifying therapy but indicates that selected Ayurvedic interventions may provide complementary benefits in symptom reduction and functional outcomes when integrated within multidisciplinary care models [28].

**Figure 3:**
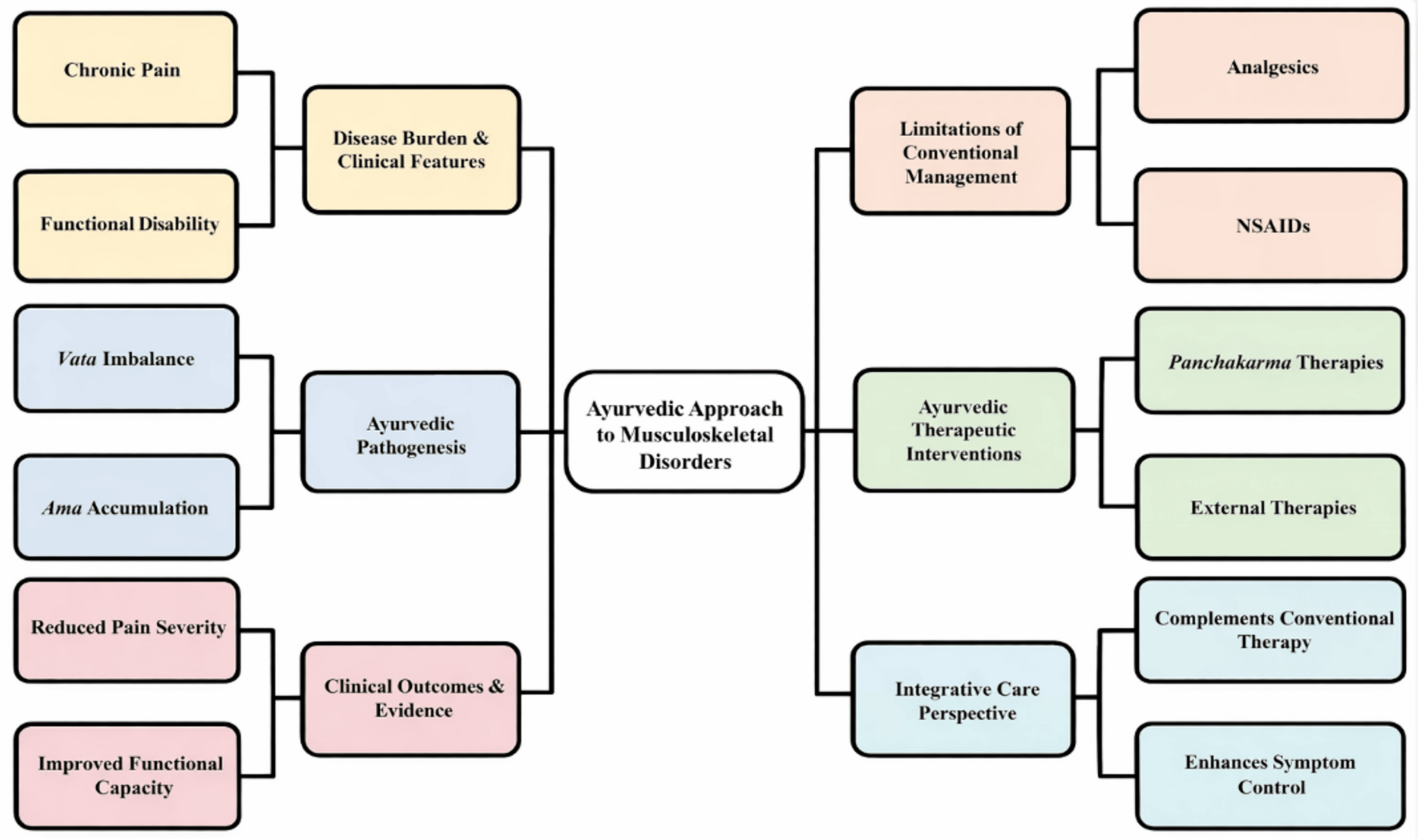
Ayurvedic management framework for musculoskeletal and rheumatic disorders This figure outlines the relationship between disease burden, Ayurvedic pathogenesis (*vata* imbalance and *ama* accumulation), limitations of conventional management, and Ayurvedic therapeutic interventions, highlighting their role in improving pain severity, functional capacity, and integrative patient care outcomes. This image was created using Microsoft PowerPoint (Microsoft Corp., Redmond, WA, USA).

Overall, while preliminary and moderate-quality clinical evidence supports symptomatic and functional improvement in selected musculoskeletal conditions, methodological variability and limited large-scale randomized trials necessitate cautious interpretation. Standardized protocols, longer follow-up periods, and clearly defined comparator-controlled designs are required to clarify the magnitude and durability of therapeutic effects. Figure 3 summarizes the integrative Ayurvedic framework applied to rheumatic and musculoskeletal disorders.

Chronic respiratory diseases and Ayurveda

Chronic respiratory diseases, including bronchial asthma, chronic obstructive pulmonary disease, and allergic airway disorders, are characterized by recurrent symptoms, periodic exacerbations, and long-term dependence on pharmacological therapy [39]. Despite advances in inhaled corticosteroids and bronchodilator regimens, approximately 5%-10% of patients with asthma have severe disease, and about 20%-50% of these patients remain uncontrolled despite optimized therapy, leading many to report persistent symptoms and concerns related to chronic corticosteroid exposure [21]. These therapeutic challenges have contributed to interest in complementary systems such as Ayurveda for long-term modulation and supportive care [34].

Within Ayurvedic classification, chronic respiratory disorders are described under Shwasa, encompassing dyspnea-predominant conditions comparable to asthma and obstructive airway disease, and Kasa, referring to persistent cough syndromes [40]. Pathogenesis is attributed to imbalance of *Vata* (regulating airflow and movement) and *Kapha* (associated with mucus production and structural stability), accumulation of *Ama*, and obstruction of respiratory channels (*Pranavaha Srotas*). This conceptual framework parallels biomedical mechanisms involving airway inflammation, bronchial hyperresponsiveness, mucus hypersecretion, and immune dysregulation [15]. Therapeutic goals therefore include reduction of airway obstruction and inflammatory burden, improvement of pulmonary function, and enhancement of immune resilience [28].

Clinical evaluation of Ayurvedic respiratory interventions has primarily focused on herbal and polyherbal formulations. *Adhatoda vasica* (Vasa) has demonstrated bronchodilatory, expectorant, and anti-inflammatory effects in clinical contexts, while *Solanum xanthocarpum* (Kantakari) has been studied for its potential role in alleviating cough, wheezing, and airway obstruction [18,41]. Polyherbal formulations such as *Sitopaladi Churna* have been associated with reductions in symptom frequency and improvement in patient-reported respiratory comfort in selected studies [33]. Some investigations report improvements in spirometric parameters, symptom severity indices, and frequency of exacerbations; however, comparator designs vary and often include adjunctive rather than standalone use [29].

Notably, certain studies suggest that Ayurvedic interventions, when integrated with conventional therapy, may contribute to improved symptom control and, in some cases, reduced corticosteroid requirements [42]. These findings indicate potential adjunctive benefit rather than replacement of standard inhaled pharmacotherapy. Nevertheless, heterogeneity in trial design, limited sample sizes, and short follow-up periods restrict definitive conclusions regarding long-term disease modification [26]. Current evidence therefore supports a possible supportive role for Ayurveda within integrative respiratory management models, while emphasizing the need for larger randomized, comparator-controlled trials with standardized pulmonary endpoints [37].

Role of Ayurveda in gastrointestinal and hepatobiliary disorders

Chronic gastrointestinal and hepatobiliary disorders, including irritable bowel syndrome, chronic constipation, and chronic liver disease, are associated with persistent symptoms, impaired quality of life, and significant healthcare utilization, with disorders of gut-brain interaction affecting up to 40.3% of adults globally and irritable bowel syndrome reported in approximately 4.1% of individuals using Rome IV diagnostic criteria [27]. These conditions frequently involve motility disturbances, inflammatory processes, metabolic dysfunction, and altered gut-liver axis regulation, and long-term therapeutic options remain limited in many cases [43].

Ayurveda conceptualizes digestive function (*Agni*) as central to both gastrointestinal and systemic health [44]. Impairment of *Agni* and accumulation of *Ama* are considered key pathological drivers, leading to disturbed digestion, absorption, and elimination [12]. *Vata* imbalance affecting intestinal motility is commonly associated with functional bowel disorders, such as irritable bowel syndrome, whereas chronic constipation is linked to weakened eliminative processes [27]. Hepatobiliary disorders are primarily attributed to *Pitta* imbalance and dysregulated metabolic transformation [45]. Therapeutic strategies focus on restoring digestive efficiency, facilitating elimination of metabolic by-products, and re-establishing gut-liver equilibrium [18].

Clinical studies evaluating Ayurvedic preparations in gastrointestinal contexts have reported improvements in both objective and patient-reported outcomes. *Triphala* has demonstrated effects on bowel regularity, abdominal discomfort, and overall digestive function in functional bowel disorders [33]. *Picrorhiza kurroa* (Kutki) has been investigated for hepatoprotective and anti-inflammatory properties, with reported improvements in liver enzyme parameters in selected populations [46]. Classical formulations such as *Arogyavardhini Vati* have been evaluated in chronic liver disease contexts, with some studies indicating symptomatic and biochemical improvement [28,47].

Most available studies emphasize symptom scales, bowel frequency measures, appetite and digestion indices, and selected biochemical markers. However, comparator designs vary widely, and many trials involve adjunctive integration with dietary regulation and lifestyle modification, making independent attribution of effects challenging [8]. Although preliminary findings suggest potential benefit in functional gastrointestinal and hepatobiliary disorders, methodological variability and limited long-term safety data necessitate cautious interpretation. More rigorously designed randomized trials with clearly defined clinical endpoints and standardized formulations are required to determine therapeutic magnitude and durability within integrative digestive care frameworks. The principal findings in gastrointestinal and hepatobiliary disorders are summarized in Table 2.

**Table 2:** Ayurvedic concepts, interventions, and clinical evidence in gastrointestinal and hepatobiliary disorders

Domain	Ayurvedic interpretation	Clinical evidence and outcomes	Reference
Disease burden	Chronic gastrointestinal and hepatobiliary disorders	High symptom persistence, impaired quality of life, and increased healthcare utilization	[43]
Core pathological factor	Impairment of *Agni* and accumulation of *Ama*	Disrupted digestion, absorption, and elimination processes	[12]
Functional bowel pathology	*Vata *dysregulation affecting gut motility	Association with irritable bowel syndrome and functional bowel disorders	[27]
Hepatic pathology	*Pitta* imbalance and altered metabolic activity	Hepatobiliary dysfunction and metabolic disturbance	[45]
Therapeutic principle	Restoration of digestive-metabolic balance	Focus on detoxification and gut-liver homeostasis	[18]
Key polyherbal therapy	Triphala	Improved bowel regularity, reduced abdominal discomfort, and better patient-reported outcomes	[33]
Hepatoprotective intervention	*Picrorhiza kurroa* (Kutki)	Reduction in liver enzyme levels and improvement in metabolic markers	[47]
Classical formulation	Arogyavardhini Vati	Improvement in hepatic function and symptom relief in chronic liver disorders	[28]
Outcome assessment	Patient-centered outcomes	Enhanced appetite, digestion, bowel habits, and overall well-being	[20]
Clinical positioning	Adjunctive role in integrative care	Supportive benefit alongside dietary and lifestyle modification	[18]

Neurological and neurodegenerative conditions

*Rasayana* therapy represents a foundational component of Ayurvedic chronic disease management, focusing on rejuvenation, systemic resilience, and healthy ageing [42]. Unlike disease-specific pharmacological interventions, *Rasayana* strategies aim to modulate broader biological processes including immune regulation, oxidative balance, metabolic efficiency, and tissue repair mechanisms [18]. This systems-oriented approach is conceptually aligned with chronic disease states characterized by progressive degeneration, inflammatory dysregulation, and functional decline [35].

From a biomedical perspective, *Rasayana* preparations have been explored primarily for immunomodulatory, antioxidant, and adaptogenic properties. Discussions of *Rasayana* in infectious contexts, including COVID-19, emphasize potential roles in host-response regulation and adaptive resilience; however, clinical outcome evidence in this area remains exploratory and largely descriptive [27]. Classical formulations such as *Chyawanprash* have been evaluated for immune-supportive and vitality-enhancing effects, particularly in elderly populations [9]. *Emblica officinalis* (Amalaki) has demonstrated antioxidant activity and possible improvement in metabolic and inflammatory parameters in selected studies [21]. *Withania somnifera* has shown improvements in stress tolerance, physical endurance, and quality-of-life measures in controlled trials [33].

Across *Rasayana* studies, improvements are frequently reported in fatigue scores, functional capacity, and patient-reported well-being [16]. Nevertheless, heterogeneity in formulation composition, dosing regimens, study duration, and outcome measures limits direct comparability. While preliminary evidence supports a potential adjunctive role in chronic disease modulation, more rigorous mechanistic studies and standardized multicenter clinical trials are required to establish reproducibility and therapeutic magnitude [40].

Safety, tolerability, and integrative approaches

Safety and long-term tolerability are critical considerations in chronic disease management, where interventions are often sustained over extended periods [7]. Clinical studies of Ayurvedic interventions generally report favorable safety profiles when therapies are appropriately prescribed and supervised, with serious adverse events infrequently documented [32]. Reported adverse effects are typically mild and transient, most commonly involving gastrointestinal discomfort [14]. However, incomplete adverse event reporting in some studies limits comprehensive safety assessment and long-term risk evaluation [26].

Challenges related to quality assurance, standardization of formulations, and potential herb-drug interactions remain important considerations [19]. Variability in raw material sourcing, preparation methods, and dosage can influence both efficacy and safety outcomes [41]. Emerging pharmacological evidence indicates that certain Ayurvedic herbs may interact with conventional medications through hepatic metabolic pathways, reinforcing the importance of integrative clinical oversight and careful monitoring [36].

Current clinical evidence predominantly supports Ayurveda as a complementary modality rather than a replacement for conventional therapy [11]. Integrative care models combining Ayurvedic and biomedical treatments have reported improvements in symptom control, patient-reported quality of life, and, in selected contexts, reduction in treatment-related adverse effects [45]. These models emphasize individualized management, interdisciplinary coordination, and context-specific application [22]. Although findings are encouraging, stronger evidence from large-scale, long-term studies incorporating standardized pharmacovigilance systems is necessary to support broader clinical implementation. An overview of safety considerations and integrative applications is presented in Table 3.

**Table 3:** Safety, tolerability, and integrative use of Ayurvedic interventions in chronic disease management

Domain	Key considerations	Clinical evidence and implications	Reference
Long-term therapy context	Chronic disease management	Safety and tolerability are critical due to the prolonged duration of therapy	[7]
Overall safety profile	Ayurvedic interventions	Assessed mainly in early-to-moderate stages of type 2 diabetes, osteoarthritis, rheumatoid arthritis, chronic stable angina, asthma, and post-viral recovery; adverse events were generally mild (e.g., gastrointestinal symptoms), with serious events rarely reported under supervised use, though long-term safety data remain limited	[32]
Common adverse effects	Mild and transient reactions	Gastrointestinal discomfort and short-term reactions are most frequently reported	[14]
Reporting limitations	Adverse event documentation	Inadequate and inconsistent reporting limit a comprehensive safety evaluation	[26]
Quality assurance issues	Standardization challenges	Variability in raw materials, manufacturing, and dosage affects safety and efficacy, although controlled trials of standardized formulations have reported improvements in glycemic control in type 2 diabetes, pain and function in osteoarthritis, lipid parameters in stable heart disease, and symptom scores in chronic respiratory disorders, with outcomes closely linked to formulation standardization and quality control	[41]
Herb-drug interactions	Pharmacological considerations	Potential metabolic and hepatic interactions with conventional drugs	[36]
Therapeutic positioning	Complementary role	Ayurveda primarily supports, rather than replaces, conventional medical care	[11]
Integrative care outcomes	Combined treatment models	In conditions such as type 2 diabetes (Ayurveda plus standard hypoglycemic therapy), osteoarthritis (herbal formulations with physiotherapy/analgesics), and bronchial asthma (adjunct herbal therapy with inhaled corticosteroids), integrative models have reported improved symptom control, better quality-of-life scores, and in some cases reduced analgesic or rescue medication use, with lower incidence of treatment-related adverse effects compared to conventional therapy alone	[45]
Care delivery model	Integrative healthcare	Emphasis on interdisciplinary collaboration and individualized care	[22]
Research priorities	Safety monitoring	Need for long-term studies with standardized pharmacovigilance systems	[47]

Limitations and future directions

Clinical studies about Ayurvedic interventions in chronic disease management have been on the rise, but the evidence is not much due to various methodological limitations. Most studies have small samples, short interventions, and little follow-up, which limits the determination of the long-term efficacy in chronic conditions. There is a lot of heterogeneity in formulations, dosages, duration of treatment and therapeutic protocols that decrease comparability between studies. Despite growing clinical interest, the existing body of evidence is constrained by methodological limitations, including inadequate randomization procedures, insufficient blinding, heterogeneous or non-standardized outcome measures, and inconsistent reporting of adverse events. These limitations affect internal validity and restrict definitive conclusions regarding long-term safety, tolerability, and comparative effectiveness, thereby necessitating cautious interpretation of available findings and underscoring the need for more rigorously designed trials.

Future studies need to be well-designed multicentric randomized controlled trials with sufficient sample sizes and longer follow-ups. Ayurvedic formulations, intervention protocols, and outcome reporting should be standardized further to enhance reproducibility and clinical translation. The combination of Ayurvedic diagnostic principles and biomedical endpoints with proven scientific rigor and interpretability could be improved. The issues of herb-drug interaction and quality control require the implementation of long-term safety evaluations and organized pharmacovigilance systems. Moreover, the use of modern research approaches, such as biomarker analysis and systems-based approaches, can enhance the evidence base and contribute to the creation of effective integrative models of chronic disease care.

## Conclusions

The present review is a synthesis of current clinical evidence on the role of Ayurveda in managing chronic illnesses and its possible role as an adjunctive and integrative healthcare method. In several chronic disease groups, such as metabolic, cardiovascular, musculoskeletal, respiratory, gastrointestinal, and neurological diseases, Ayurvedic treatments show similar tendencies in reduction in the burden of symptoms, functional outcomes, and quality of life as measured by the patient. The Ayurveda approach to chronic diseases is multifactorial and long-term; therefore, the holistic and personalized approach of Ayurveda, which involves metabolic regulation, lifestyle change, psychosomatic balance, and *Rasayana*-based rejuvenation, is quite consistent with the nature of chronic diseases. There is emerging clinical evidence that Ayurvedic therapies can have anti-inflammatory, antioxidant, immunomodulatory, and adaptogenic effects of interest to the pathophysiology of chronic diseases. Nevertheless, the existing evidence base has methodological heterogeneity, inconsistent quality of studies, and a lack of long-term safety data, which require cautious interpretation of the results. Notably, the literature confirms the application of Ayurveda as a complementary therapy in addition to conventional medicine instead of an alternative. Further development of Ayurveda in chronic disease treatment will need highly structured clinical trials, standard treatment procedures, and well-developed safety monitoring systems to enhance clinical confidence and translational usability.
